# Exploiting the mechanism of estrogen-induced transcription to fight breast cancer

**DOI:** 10.1038/s12276-021-00656-1

**Published:** 2021-07-29

**Authors:** Bruno Perillo, Gabriella Castoria, Antimo Migliaccio

**Affiliations:** 1 Istituto per l’Endocrinologia e l’Oncologia Sperimentale “G. Salvatore”, C.N.R., via S. Pansini, 5, 80131 Naples, Italy; 2 Dipartimento di Medicina di Precisione, Università della Campania “L. Vanvitelli”, via L. De Crecchio, 7, 80138 Naples, Italy

**Keywords:** Base excision repair, Histone post-translational modifications

**Fig. 1:**
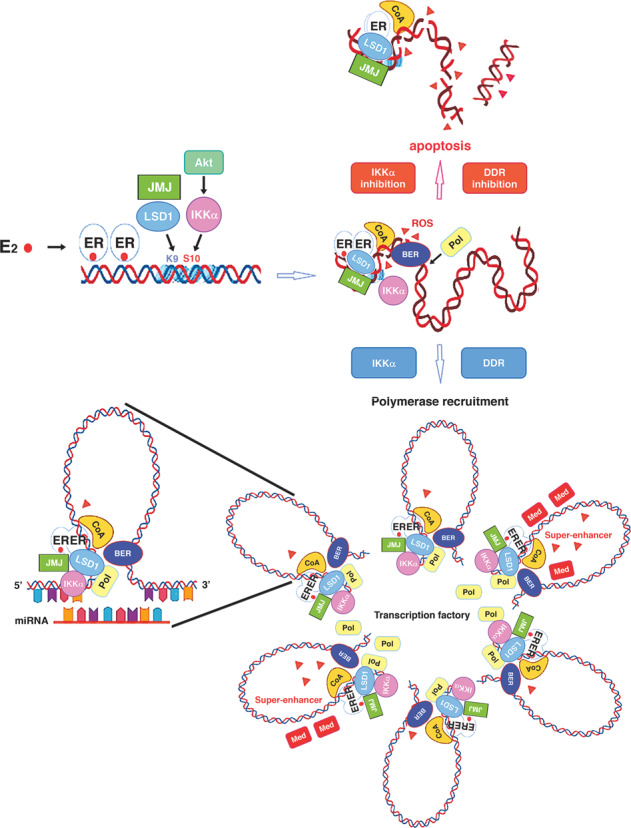
Estrogen-(E2)-bound receptor (ER), together with coactivators (CoA), drives removal of methyl marks from H3 lysine 9 (K9) by demethylases (LSD1 and JMJ). Meanwhile, Akt, through the IKKα kinase, triggers phosphorylation of H3 serine 10 (S10), providing the safeguard mechanism described in the text. H3K9 demethylation generates ROS (red triangles) that induce DNA nicks by the base excision repair (BER) enzymes, creating the conditions for gene looping between the 5′ and 3′ ends upheld by untranslated micro-RNAs (miRNA). Several genes converge in transcription factories where can fall under the control of the super-enhancers that show a relevant accumulation of master transcription factors (here exemplified by the mediator complex Med). As defense by ROS, cells line up the DNA damage repair (DDR) response apparatus that, if fails because specifically inhibited (DDR inhibition) or overwhelmed by excessive ROS (IKKα inhibition), triggers cell death by apoptosis.

Dear Editor,

Thousands of responsive genes react in a coordinated fashion to estrogen challenge by creating loops supported by small untranslated RNAs that, similar flower petals, converge at the center of the gene transcription machinery (see Fig. 1)^1–3^. In this context, several genes are controlled by a novel class of enhancers, called superenhancers (SEs), that enable significant responses of regulated genes (see Fig. 1)^4^. Jia et al. reviewed the role of SEs in tumorigenesis and characterized oncogenic superenhancers that, originating from mutations, chromosomal rearrangements or spatial alterations, generate aberrant signaling pathways and play roles in the initiation and progression of tumors^5^. In their review, this group also identified oncogenic SEs as promising targets for anticancer treatment strategies whose crucial challenge is the identification of selective inhibitors^5^.

Interestingly, the focus of transcriptional activity at discrete areas leads to a high chromatin torsional stress that requires an efficient DNA damage repair (DDR) response powered by a DDR-dedicated protein network. Wengner et al. reviewed alterations in DDR enzymes in hormone-dependent cancer cells that promote mutations and/or gene expression changes that drive the initiation and progression of these cancers^6^. The authors also described the beneficial effects of several DDR inhibitors for the treatment of prostate and breast cancer, especially in conjunction with agents that enhance DNA damage^6^. The same conclusions were reported in a recent review that highlights DDR as a therapeutic target for patients with pancreatic cancer, showing that new strategies based on the use of DNA-damaging agents in conjunction with inhibitors of poly (ADP-ribose) polymerase, a representative DDR enzyme, show encouraging results^7^.

We previously showed that hormone-regulated gene transcription is triggered by estrogen receptor alpha (ERα)-dependent demethylation of lysine 9 in H3 histone (H3K9) catalyzed by specific demethylases^8,9^. Notably, demethylation of H3K9 is followed by generation of reactive oxygen species (ROS) that elicit oxidation of adjacent guanine residues, which are recognized by base-excision repair enzymes that induce DNA breaks, allowing DNA unrolling and recruitment of transcriptional machinery (see Fig. 1)^8^. Thus, transcription is a ROS-related DNA stress-inducing process that requires an efficient repair response to prevent programmed cell death (PCD). In fact, the activation of different pathways that increase intracellular ROS levels has been intensively explored as an inducer of cancer cell apoptosis^10^.

To prevent excessive ROS production during transcription, a safeguard mechanism represented by phosphorylation of H3 histone at serine 10 has evolved to block rapid remethylation of the preceding lysine and downregulate methylation/demethylation cycles, allowing the scavenging of such molecules^11^. Therefore, inhibition of this control mechanism likely results in the overproduction of ROS with the induction of PCD, and transcription at designated factories, even though undoubtedly efficient, may be considered a double-edged sword, with the cellular Achille’s heel in the accumulation of local concentrations of ROS that are much higher than those in the surrounding space.

Hence, we imagine that PCD can be triggered in breast cancer cells by employing treatments that damage DNA through the overproduction of transcription-dependent ROS induced by inhibitors of enzymes involved in the self-regulatory mechanisms elicited by hormone challenge, with the effect exacerbated by the concurrent inhibition of the DDR response. Interestingly, our strategy can be applied to androgen-responsive tumors and to triple-negative breast cancers that express the retinoic acid receptor.
